# Effects of traditional harvest and burning on common camas (*Camassia quamash*) abundance in Northern Idaho: The potential for traditional resource management in a protected area wetland

**DOI:** 10.1002/ece3.8010

**Published:** 2021-09-01

**Authors:** Devin S. Stucki, Thomas J. Rodhouse, Ron J. Reuter

**Affiliations:** ^1^ National Park Service, Inventory and Monitoring Program Bend Oregon USA; ^2^ Department of Forest Ecosystems and Society Oregon State University–Cascades Bend Oregon USA

**Keywords:** geophyte, restoration, traditional ecological knowledge, traditional resource management, wetland

## Abstract

The bulbs of common camas (*Camassia quamash*) were a staple food of Indigenous Peoples of western North America for millennia. Camas harvesting site productivity was encouraged through intense management. Common camas is considered a facultative wetland species, and populations have declined due to contemporary wetland drainage and land conversion. Conservation of existing habitat, as well as restoration of degraded systems, is necessary. Traditional ecological knowledge (TEK) and resource management (TRM) are often promoted as viable modes of contemporary resource management but are rarely tested or implemented. We designed a controlled experiment, informed by a born‐in‐the‐tradition specialist, to evaluate the response of common camas populations to traditional bulb harvest, burning, and a combination of harvest and burning. We recorded camas plant counts of three life stage classes of camas plants (single‐leaf seedling, multiple‐leaf adult, and flowering adult) over the course of 6 years in arrays of plots and subjected to treatments or left undisturbed as control. Harvesting removed plants (>800 bulbs) and reduced aboveground counts of camas densities ($\bar{X}$ ~ 50% of control, *p* < .05). Burning contributed to a reduction in single‐leaf plants but had an overall positive effect ($\bar{X}$ ~ 150% of control, *p* < .05) on adult camas and flowering plant abundance, and ameliorated the digging impacts. Treatment impacts tapered over the course of the study, and results indicate that a sustainable harvesting return interval of approximately 5 years may be possible when combined with fire to reduce litter and competition from pasture grasses and to accelerate the recovery of camas. Our findings support the hypotheses proposed by traditional knowledge specialists and ethnobotanists that digging and burning reduce intra‐ and interspecific competition and stimulate the growth of unharvested adult plants. More generally, our study supports the integration of traditional ecological knowledge into the evidence base available for protected area wetland prairie management.

## Graphical abstract

Common camas (*Camassia quamash*), a native geophyte with an edible bulb, was a staple food of Indigenous Peoples of the Pacific Northwest, and camas harvesting sites were intensely managed to encourage productivity. This article summarizes the results of an experiment conducted to assess the effects of traditional harvest and burning on common camas populations and discusses the use of traditional resource management for camas prairie restoration.

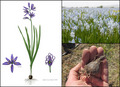

## 1 INTRODUCTION

Wetland habitats declined dramatically across what is now the United States of America (USA) following colonization in the 1780s (Dahl, 1990), and these declines have continued into the twenty‐first century mainly due to anthropogenic impacts, particularly wetland drainage and conversion (Claassen et al., 2011; Dahl, 2011; Davidson, 2014, 2018). Although wetland conversion in the USA has slowed in recent years (Dahl, 2011; Davidson, 2014), reduction in wetland quality, impacted wetland dynamics, and invasion by exotic species remain major threats to wetland biodiversity (Dudgeon et al., 2006; Kingsford et al., 2016; Mitsch & Hernandez, 2013; Zedler & Kercher, 2005 ).

Conservation of intact wetland habitat is necessary where possible, but restoration may be needed in habitats that are degraded or where a stressor has led to the decline of a species or system. Many restoration approaches come with large financial costs and may not be well suited for certain species or systems (Mitsch & Wilson, 1996), particularly in protected areas with a historical or cultural context that must be maintained (Anderson & Barbour, 2003; Keenleyside et al., 2012). Restoration efforts that reintroduce historical disturbances have grown in favorability in recent years, in part because reintroduced processes (e.g., disturbance) may improve the chances for success with desired state changes (e.g., species establishment or community composition) (Anderson & Barbour, 2003; Storm & Shebitz, 2006; Uprety et al., 2012). The incorporation of traditional ecological knowledge and resource management practices into contemporary ecological restoration is one approach that has been used to guide and implement these historical disturbances (Anderson & Barbour, 2003; Lertzman, 2009; Zedler & Stevens, 2018).

The (typically) small‐scale disturbances caused by many traditional societies in association with food harvest and agroforestry practices can promote ecosystem renewal (Berkes et al., 2000; Turner, 1994; Turner et al., 2013). Traditional resource management (TRM), which draws on traditional ecological knowledge (TEK) and practices, may be well suited for systems where a group of people have a strong cultural tie to a species or community of concern that may benefit from some specific disturbance (Anderson & Barbour, 2003; Berkes et al., 2000). While TRM may be an important and useful toolset for restoration in some wetlands, these practices are rarely tested or implemented, possibly because the methods are poorly understood by land managers, and traditional knowledge specialists are not engaged with or given access to participate in contemporary conservation science (Kimmerer, 2002; Senos et al., 2006). Lertzman (2009) and Zedler and Stevens (2018) establish clear parallels between the methods and objectives of TRM and western, science‐based land management practices, and contend that the incorporation of TRM may be vital to understanding all facets of a particular restoration challenge. Decolonizing ecological expertise, in part by elevating the role of Indigenous knowledge and participation in decision‐making, provides alternative practices and management strategies while helping to build inclusiveness and legitimacy in decision‐making (Trisos et al., 2021).

Common camas lily (*Camassia quamash* (Pursh) Greene) is considered a functional wetland plant over much of its range (Lichvar et al., 2016; U.S. Army Corp of Engineers, 2018), though it can be found growing in a variety of habitats such as oak savannas and scablands. Common camas (hereafter camas) is a bulb‐forming geophyte that can grow in dense colonies in wet prairie ecosystems, many of which have been drained or otherwise degraded from agriculture, livestock grazing, and weed invasion. Camas has been a staple food for Indigenous Peoples of Northwestern USA and SW Canada for millennia (Hunn, 1981; Mastrogiuseppe, 2000; Thoms, 1989; Turner & Kuhnlein, 1983) and hundreds of metric tons of the edible bulbs were harvested annually across the region (Thoms, 1989). The anthropogenic disturbances associated with digging and harvesting, which include the removal of competing and/or undesirable vegetation (such as death camas, *Toxicoscordion venenosum*) and sometimes post‐harvest burning, have been reported both by traditional knowledge specialists as well as contemporary science to intensify camas populations, increase the size of camas bulbs, and create growing conditions more suitable for mature plants as well as the germination of camas seeds (Anderson, 1997; Beckwith, 2004; Boyd, 1999; Hamman et al., 2011; Kramer, 2000; Stevens et al., 2000; Storm & Shebitz, 2006; Suttles, 1951; Thoms, 1989). Digging for camas bulbs introduces organic matter into the soil (Thoms, 1989) provides soil aeration and mixes soil components (Beckwith, 2004; Harris, 1989) and results in nutrient exchange (Harris, 1989; Thoms, 1989). Because smaller bulbs are returned to the soil (Thoms, 1989; Turner & Kuhnlein, 1983), plants are available to recover a harvested site. Both bulb harvest and post‐harvest burning reduce litter and thatch and provide bare‐soil microsites, conditions more suitable for seed germination and seedling elongation (Anderson, 1993; Boyd, 1999; Stevens et al., 2000; Storm & Shebitz, 2006). Burning also keeps meadows and clearings open by eliminating encroaching trees and shrubs and may aide in beneficial nutrient exchange (Anderson, 1993; Boyd, 1999; Storm & Shebitz, 2006).

To test the traditional knowledge hypothesis that harvesting camas bulbs and burning vegetation within the harvesting site would increase camas plant densities, we evaluated the response of camas plants to bulb harvest, burning, and a combination of both harvest and burning, in 50 permanent 1 m^2^ experimental plots located among a wild common camas population in Weippe Prairie National Historic Landmark, Idaho (hereafter Weippe Prairie NHL), a protected area managed by the National Park Service Nez Perce National Historical Park. Here, we present the results of camas density responses (counts) for seedlings, mature plants, and flowering plants to these experimental treatments.

Two other unpublished experiments have been conducted to assess the effects of camas bulb harvest (Beckwith, 2004; Proctor, 2013), though both studies focused on either mixed populations of great camas (*Camassia leichtlinii* (Baker) S. Watson) and common camas, or great camas alone. These studies were also both conducted on Vancouver Island, BC, an area with a maritime climate quite different from the continental climate experienced in the Rocky Mountains where Weippe Prairie NHL is located. These two other studies did, however, use similar sampling methods and response variables, and their results provide relevant context for our own. Our study will be an important addition to the current understanding of TRM of camas plants. More specifically, this research documents how a culturally important and ecologically threatened species reacts to these types of disturbances, important information for land managers tasked with conserving camas and facilitating traditional harvest on their lands.

## 2 METHODS

### 2.1 Species description

**FIGURE 1:**
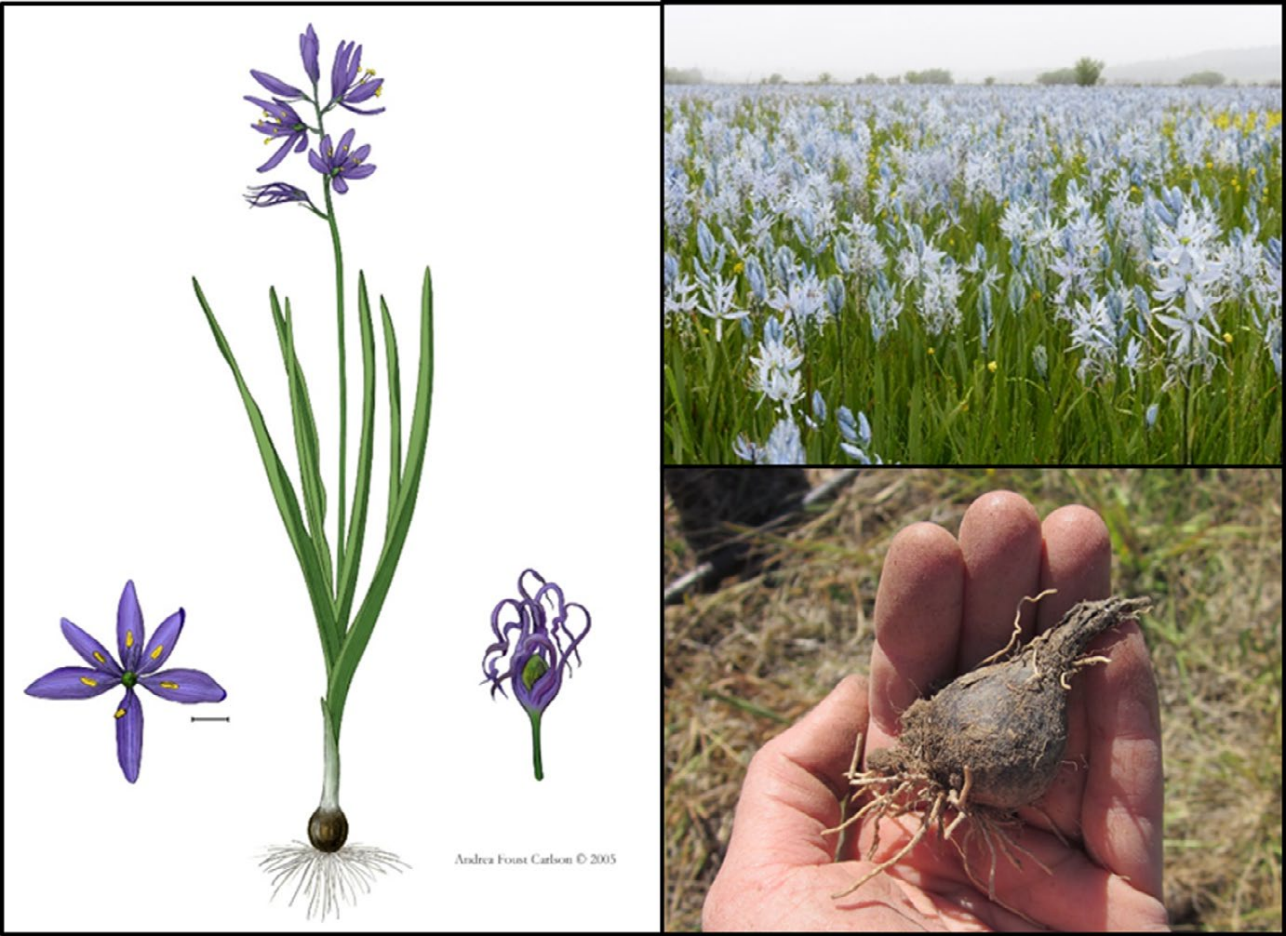
(left) Illustration of *Camassia quamash* (Pursh) Greene. Reproduced with permission from Andrea Foust Carlson. (upper right) Dense fields of *C. quamash* growing at a study site in Weippe Prairie, Idaho. (lower right) Harvested *C. quamash* bulb in hand

Camas is a long‐lived (20–30 years; Thoms, 1989), polycarpic, bulbous geophyte with showy blue flowers (Figure 1; Gould, 1942; Maclay, 1928; Ranker & Hogan, 2002). It occupies a variety of habitats including seasonally wet meadows and prairies, oak savannas, rocky bluffs, and scablands from near sea level to over 3,300 m above sea level throughout the Pacific Northwest, Sierra Nevada mountains, and Northern Rocky Mountains in North America (Beckwith, 2004; Reed, 1988; Thoms, 1989; Turner & Kuhnlein, 1983). Camas is capable of asexual reproduction through bulb division (Genders, 1973; Maclay, 1928) though rarely does so in the wild (Beckwith, 2004; Thoms, 1989; D. Stucki, pers. obs.) and instead primarily reproduces sexually by seed (Thoms, 1989), relying on a variety of insect pollinators (Bartow, 2015; Pendergrass et al., 2008).

Temperate geophytes including camas produce aboveground growth from spring through early summer (de Hertogh & le Nard, 1993; Maclay, 1928). The aboveground vegetative growth senesces in summer and the plant exists as an underground storage organ, a bulb in the case of camas, until the following spring. Camas plants can remain dormant underground or abort aboveground growth when growing conditions are unfavorable (Beckwith, 2004; Thoms, 1989; Tompsett, 1985). A closely related species, *C. leichtlinii,* was found to have a 10% dormancy rate in bulbs following transplanting, with the rate of dormancy decreasing over the course of the study period (Beckwith, 2004). A rate of dormancy was not provided for common camas, though Beckwith (2004) found that some common camas bulbs remained dormant for up to three growing seasons following transplanting before producing aboveground vegetation.

For this study, we categorized camas plants using leaf‐number and reproductive status as indicators of three life stages: single‐leaf (immature), multiple‐leaf (mature), and flowering plants (reproductive). The decision was consistent with the long‐term camas monitoring protocol developed by the National Park Service (NPS) and in use at Weippe Prairie NHL (Rodhouse et al., 2011; Rodhouse et al., 2007). Camas seedlings emerge with a single true leaf, while harvestable‐sized camas bulbs usually have multiple leaves (Bailey, 1914; Genders, 1973; Thoms, 1989). Monitoring of camas plant densities by counting aboveground basal leaves provides a useful estimate of the number of belowground harvestable‐sized bulbs, though, Beckwith (2004) noted that because an unknown number of camas plants may be dormant during counting, using plant counts as a proxy for bulb counts likely results in a conservative estimate.

### 2.2 Study site

**FIGURE 2:**
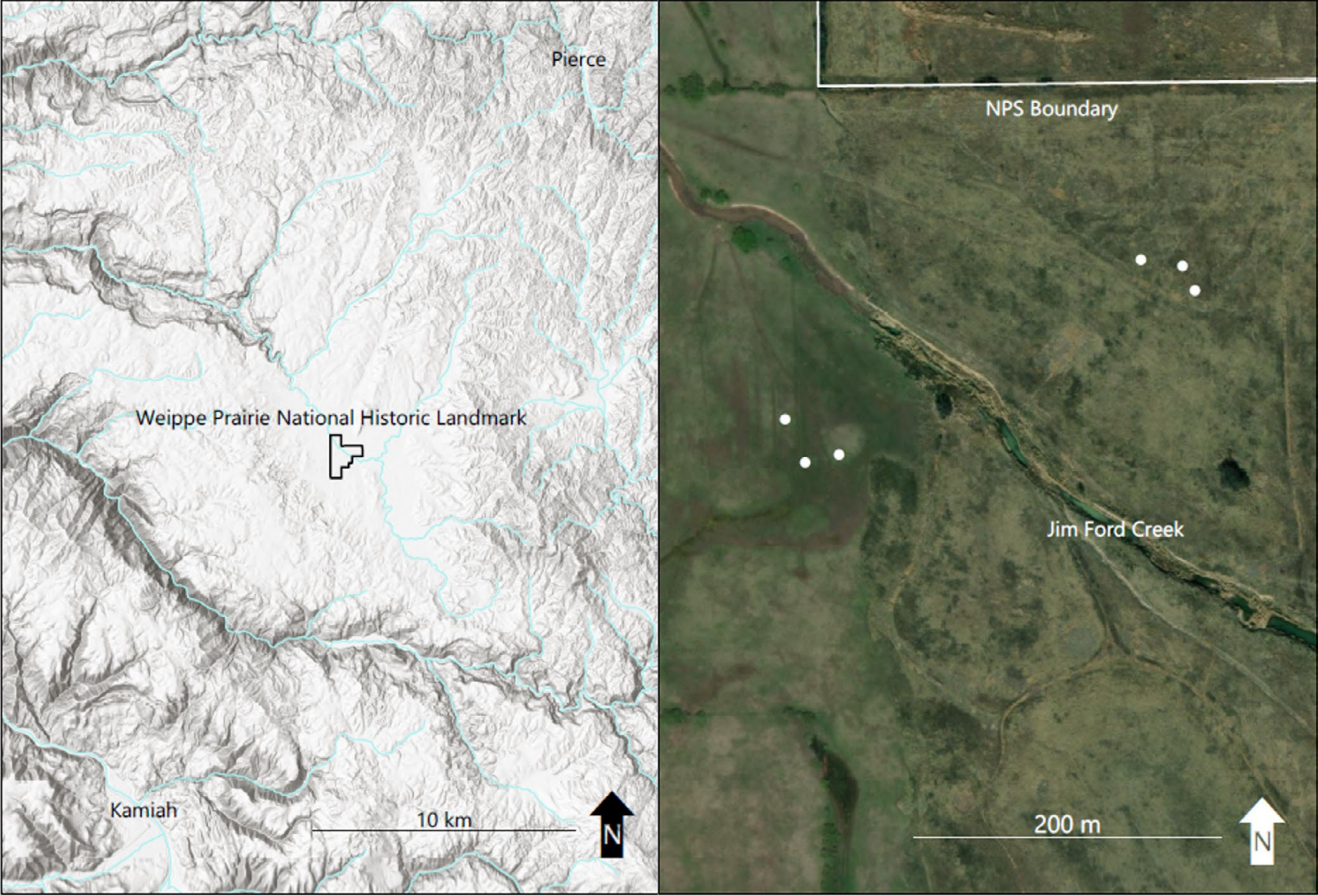
Map of the study area. (left) Hill‐shade relief image showing location of Weippe Prairie National Historic Landmark on the broad plain of Weippe Prairie, east of the Clearwater River. (right) Magnified view of the study area with approximate park boundaries and significant waterways outlined in light‐gray, and plot array locations symbolized with white circles

Weippe Prairie, located on a broad plain above the Clearwater River in Northern Idaho, USA (Figure 2), was a traditional camas harvesting ground and gathering place for the Nez Perce tribe (Ames & Marshall, 1981; Marshall, 1977). The area was a vital resource for the Nez Perce people due to the high densities of camas that historically grew there. Weippe Prairie is the location where the Nez Perce first encountered the Lewis and Clark expedition in 1805, and where Meriwether Lewis collected the holotype for *C. quamash* (Gould, 1942; Pursh, 1814). One hundred ten ha are designated as Weippe Prairie National Historical Landmark as part of the Nez Perce National Historical Park managed by the NPS. Soils at Weippe Prairie NHL are dominated by alluvial and lacustrine sediments overlain with loess and volcanic ash, are fine in texture, and composed of smaller particle sizes including silt and clay (McDaniel & Falen, 2011, 2014). Soils are perched on slowly permeable subsoil horizons across a low‐lying plain with gentle topography and are inundated by water during the winter and spring months (McDaniel & Falen, 2011). Native vegetation at the site is composed of functional wetland forbs and graminoids, and black hawthorn trees (*Crataegus douglasii*). Weippe Prairie NHL is also dominated by non‐native European pasture grasses such as timothy grass (*Phleum pratense*) and meadow foxtail (*Alopecurus pratensis*) and is host to the highly invasive reed canary grass (*Phalaris arundinacea*), which is actively being addressed by Weippe Prairie NHL managers but represents a threat to camas populations at the site if left unchecked.

Following European settlement in the late 1800s, much of the site was homesteaded, converting the seasonal wetlands into farmland. Homesteading effectively put an end to traditional camas harvest in Weippe Prairie, as traditional camas harvesting grounds that had been passed down over centuries were claimed by European homesteaders and converted into farmland (Marshall, 1977). Weippe Prairie NHL is made up of several parcels of land, each with a varied history of agricultural uses, including pasture for livestock and haying. Much of the site was dissected with drainage ditches to improve pasture for livestock, and the effects of drying the prairie are still present (McDaniel & Falen, 2011). Despite the past history of land management at Weippe Prairie NHL, the site supports a large population of camas plants, and NPS camas monitoring results show that camas plant densities, while annually variable, have remained stable and increased in some cases, likely in part due to cessation of haying and livestock grazing, and ongoing invasive plant control (Rodhouse et al., 2011; Stucki & Rodhouse, 2017).

### 2.3 Experimental design

Strategic but non‐random placement of plot arrays was required in Weippe Prairie NHL due to several management concerns including drainage ditches and canals, Jim Ford Creek and associated riparian area, dense stands of hawthorn trees, and large areas with little or no camas plants. We used the results of long‐term camas plant monitoring data (Rodhouse & Stucki, 2013) to locate areas of continuous medium to high camas density (approximately 20 multiple‐leaf plants/m^2^ or greater) in an attempt to reduce variability in camas counts and to ensure that all plots contained at least some camas plants, though actual camas density varied widely between plots.

Plots were placed within areas previously mapped as having similar soil types in order to reduce the variability of camas density (McDaniel & Falen, 2011). Areas disturbed by vehicles, recently treated for noxious weeds, or otherwise heavily disturbed were avoided, as were those identified for current and future restoration or research projects by the Nez Perce National Historical Park Resource Management. Areas infested by invasive plants were also avoided, and Park management agreed to suspend herbicide application within the immediate vicinities of the plots during the study.

We established 50 experimental plots (each 1 m^2^) in Weippe Prairie NHL. Baseline data were collected for each plot during the year of establishment. Each plot received a single treatment only once during the year of establishment, with post‐treatment data collected during annual plot revisits. In 2013, 34 plots were established, arranged in four arrays of eight plots each (2 rows of 4 plots in each array), with two additional plots added to one of the arrays. Each plot established in 2013 was randomly assigned one of four treatment types: a digging and bulb harvesting treatment, a burning treatment, a combination treatment of digging and harvesting followed by burning, or no treatment (control). In 2014, another 16 plots were established, this time in two arrays of eight plots (2 rows of 4 plots) each. Two treatment types were assigned to plots; digging and harvesting of camas bulbs, and no treatment (control). Traditional digging and harvesting were chosen as the sole treatment during the second year of the study because park management considered it a priority to evaluate the effects of harvest. Plot treatments were randomly assigned to the first plot in each array, then treatment assignments alternated between each subsequent plot. This approach provided unbiased treatment assignment while maintaining spatial balance between treatment types.

### 2.4 Traditional harvest methods

**FIGURE 3:**
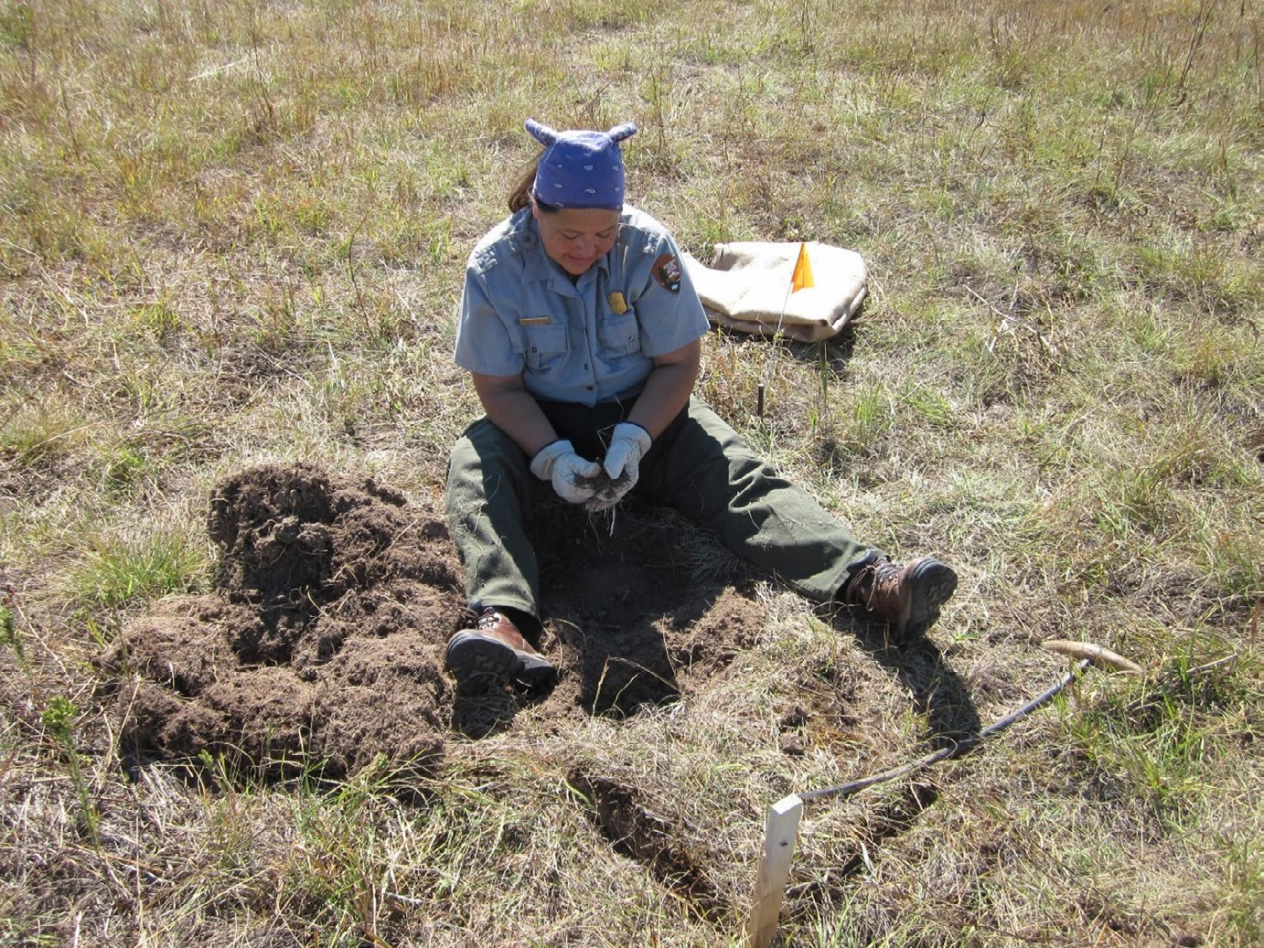
Vivian Wilson, Nez Perce National Historical Park, harvesting camas bulbs within a 1 m^2^ experimental plot at Weippe Prairie National Historic Landmark, Idaho. Note the traditional curved digging stick, called a *tú·kes*, in the foreground

Digging and harvesting treatment methods followed those outlined in an unpublished manuscript describing the process of harvesting camas bulbs with Nez Perce traditional gatherers Lee Bourgeau and her daughter Kamelle Bourgeau, at Weippe Prairie NHL (J. Jocius, unpublished; see Supplementary Material). The process is described as follows: After a suitable area with available camas was located, the layer of sod and thatch was loosened using a curved digging stick called a *tú·kes*, or a more modern tool called a spading fork (Figure 3). Clumps of sod were turned over, and dense clods of soil were broken apart by hand to reveal the camas bulbs within. Bulbs about the size of a person's thumbnail or larger (approximately 15 mm in diameter) were harvested while smaller bulbs were set aside to be replaced in the ground when harvest was concluded for that particular area. Digging continued outwardly in the direction of available camas bulbs, causing the shape and size of the hole to change according to adjacent camas densities. When availability of harvestable camas bulbs declined in the immediate vicinity, the hole was refilled with previously removed soil, surface vegetation, and smaller camas bulbs. This resulted in smaller bulbs being distributed at different depths throughout the freshly dug soil profile. Most camas seeds had already fallen to the ground from the dehiscent capsules, and no particular effort was made to distribute them into the hole, though physical disturbance caused by digging caused some seed to fall.

### 2.5 Plot treatments

During both years of plot establishment, plot treatments were carried out in mid‐September, very near the date of September 20 when the Nez Perce were observed harvesting camas in Weippe Praire by the Lewis and Clark Expedition (Marshall, 1977; Sappington, 1989). Burning was historically carried out during the same year and often immediately following harvest treatments (Storm & Shebitz, 2006; Thoms, 1989). Plot treatments were applied during the year of plot establishment only and each plot received only one treatment. Subsequent plot revisits were necessary solely to assess treatment effects.

Treatments were designed to closely mimic the traditional bulb harvest methods described above (Figure 3). During the harvest treatment, the sod layer within plots was loosened and turned over, and camas bulbs were removed by hand. Digging proceeded by loosening the soil with a *tú·kes* to the maximum depth of camas growth, approximately 25 cm, during which clumps of soil were broken up by hand and harvestable‐sized (>15 mm diameter) camas bulbs were retained. After digging to the edges of the plot and thoroughly searching for harvestable camas bulbs, all smaller bulbs were replaced back into the hole, along with the previously removed soil and sod.

Plots treated with burning, and a combination of harvest and burning, were surrounded with a custom‐made 1 m^2^ fire box, similar to that reported by Korfmacher et al. (2003). This design provided adequate airflow while containing firebrands and enabled uniform combustion of aboveground litter and vegetation. Litter and vegetation within plots were ignited with a propane torch and allowed to burn unaided until active flames had receded. Flames were allowed to smolder and die out naturally without disturbance or addition of water. Plots treated with a combination of harvest and burning were first dug and harvested according to the methods above, then the remaining vegetation within plots was burned after the soil and sod had been replaced. Organic matter within burned plots only appeared to be impacted within the top 5 cm of soil, and aboveground vegetation was charred but not vaporized, suggesting that fire intensity was low, though it was not directly measured.

### 2.6 Data collection

We established baseline counts of three life stage classes of camas plants, including single‐leaf, multiple‐leaf, and flowering camas plants, a decision consistent with previous studies (Bailey, 1914; Beckwith, 2004; Thoms, 1989) and compatible with methods outlined for NPS long‐term camas monitoring by Rodhouse et al. (2007), Rodhouse et al. (2011). Following plot treatments, camas counts were recorded on an annual basis over the study period of 2013–2019 (no data were collected in 2017), providing either 5 or 6 years of post‐treatment data, depending on the year of plot establishment. We counted camas plants during late‐May when the plants are in bloom at Weippe Prairie NHL.

### 2.7 Data analysis

We constructed generalized linear mixed‐effect models (GLMM; McCulloch & Searle, 2001) to assess the strength of plot treatment effects on camas density within three life stage classes, including single‐leaf, multiple‐leaf, and flowering camas plants.

We modeled camas plant counts using a negative binomial link because of overdispersion in counts (i.e., variance > mean; Hilbe, 2011; Stroup, 2014). Our model was structured as:

$$\log\left(\mu \right)=\beta_{0}+\beta_{1}*\text{Tx}+\beta_{2}*\text{Year}+\beta_{3}*{\text{Year}}^{2}+\beta_{4}*\text{Tx}*\text{Year}+\beta_{5}*\text{Tx}*{\text{Year}}^{2}+\alpha_{0,\text{array}}+\alpha_{1,\text{array}}*{\text{Year}}_{\text{array}},$$

where *β*_0_ represents the mean count (*μ*), after accounting for covariates, and where Tx is an indicator variable for treatment group. Note that year + year^2^ allows for a quadratic (curvilinear) relationship between count and time, and Tx * year + Tx * year^2^ allows for an interaction between treatment group and time. The year was centered around the mid‐point of the study period (2016). Random effects are represented by *α*, allowing both intercepts and slopes through time to vary by array membership (Gelman & Hill, 2007). This construction helps to account for the potential lack of independence among plots nested within arrays (i.e., plots within arrays are likely to be more similar than plots from different, widely separated, arrays).

All statistical analyses were conducted in R version 3.6.1 (R Core Team, 2019). We constructed GLMMs using the function glmmADMB in the package glmmADMB (Fournier et al., 2012). Standard errors, *p* values, and 95% confidence intervals were generated for each model and used to evaluate the strength of evidence for treatment effects (Table 1).

**TABLE 1:** Results of generalized linear mixed models for 3 life stage classes of camas density counts showing estimated effects of treatment and time on single‐leaf, multiple‐leaf, and flowering camas plants

	Single‐leaf camas plants				Multiple‐leaf camas plants				Flowering camas plants			
	Estimate	CI	*SE*	*p*	Estimate	CI	*SE*	*p*	Estimate	CI	*SE*	*p*
Intercept	205.33	120.74–349.17	55.62	**<.001**	68.13	43.80–105.96	15.35	**<.001**	14.38	8.62–23.99	3.75	**<.001**
Year	1.01	0.94–1.07	0.03	.834	0.92	0.86–0.98	0.03	.**011**	0.87	0.79–0.95	0.04	.**002**
Year^2^	1.00	0.97–1.03	0.02	.830	1.02	0.99–1.06	0.02	.198	0.99	0.94–1.03	0.02	.544
Burn	0.94	0.71–1.24	0.13	.653	1.42	1.03–1.95	0.23	.**032**	1.75	1.16–2.66	0.37	.**008**
Dig	0.73	0.58–0.91	0.08	.**005**	0.60	0.46–0.77	0.08	**<.001**	0.35	0.25–0.50	0.06	**<.001**
Dig/Burn	0.53	0.40–0.70	0.08	**<.001**	0.83	0.60–1.14	0.13	.249	0.86	0.57–1.30	0.18	.470
Year * Burn	1.05	0.97–1.14	0.04	.225	1.05	0.96–1.16	0.05	.305	1.11	0.98–1.26	0.07	.110
Year * Dig	0.93	0.87–1.00	0.03	.057	1.00	0.92–1.08	0.04	.993	0.97	0.87–1.08	0.05	.590
Year * Dig/Burn	1.07	0.98–1.16	0.05	.122	1.01	0.92–1.11	0.05	.885	1.04	0.92–1.19	0.07	.521
Year^2^ * Burn	1.01	0.96–1.06	0.03	.765	0.97	0.91–1.02	0.03	.252	0.93	0.86–1.00	0.04	.056
Year^2^ * Dig	1.05	1.00–1.09	0.02	.**039**	1.06	1.01–1.12	0.03	.**017**	1.12	1.04–1.19	0.04	.**002**
Year^2^ * Dig/Burn	1.07	1.02–1.13	0.03	.**007**	1.00	0.94–1.06	0.03	.920	0.98	0.91–1.05	0.04	.528

Note

Statistically significant results with 95% confidence intervals that do not include 0 and with *p*‐values < .05 are shown in bold text.

## 3 RESULTS

### 3.1 Camas counts

Camas counts were highly overdispersed, as expected, and varied widely within life stage classes and among plots (see Table 1), despite initial attempts to reduce variability by locating plots in areas of continuous camas density. Camas plant counts per plot ranged from 9 to 980 per m^2^ for single‐leaf plants, 5 to 291 per m^2^ for multiple‐leaf plants, and 0 to 94 per m^2^ for flowering plants. The number of harvested bulbs in plots treated with traditional harvesting ranged from 1 to 110 per m^2^ and averaged 35 bulbs per plot (total of 863 bulbs removed during treatments). The average diameter of harvested bulb was 2.2 cm (*SD* 0.4 cm), and bulbs ranged from 1.5 to 4.0 cm in diameter, similar to the bulb sizes reported for common camas by Hebda (1992) and Beckwith (2004).

### 3.2 Treatment effects on single‐leaf camas plants

**FIGURE 4:**
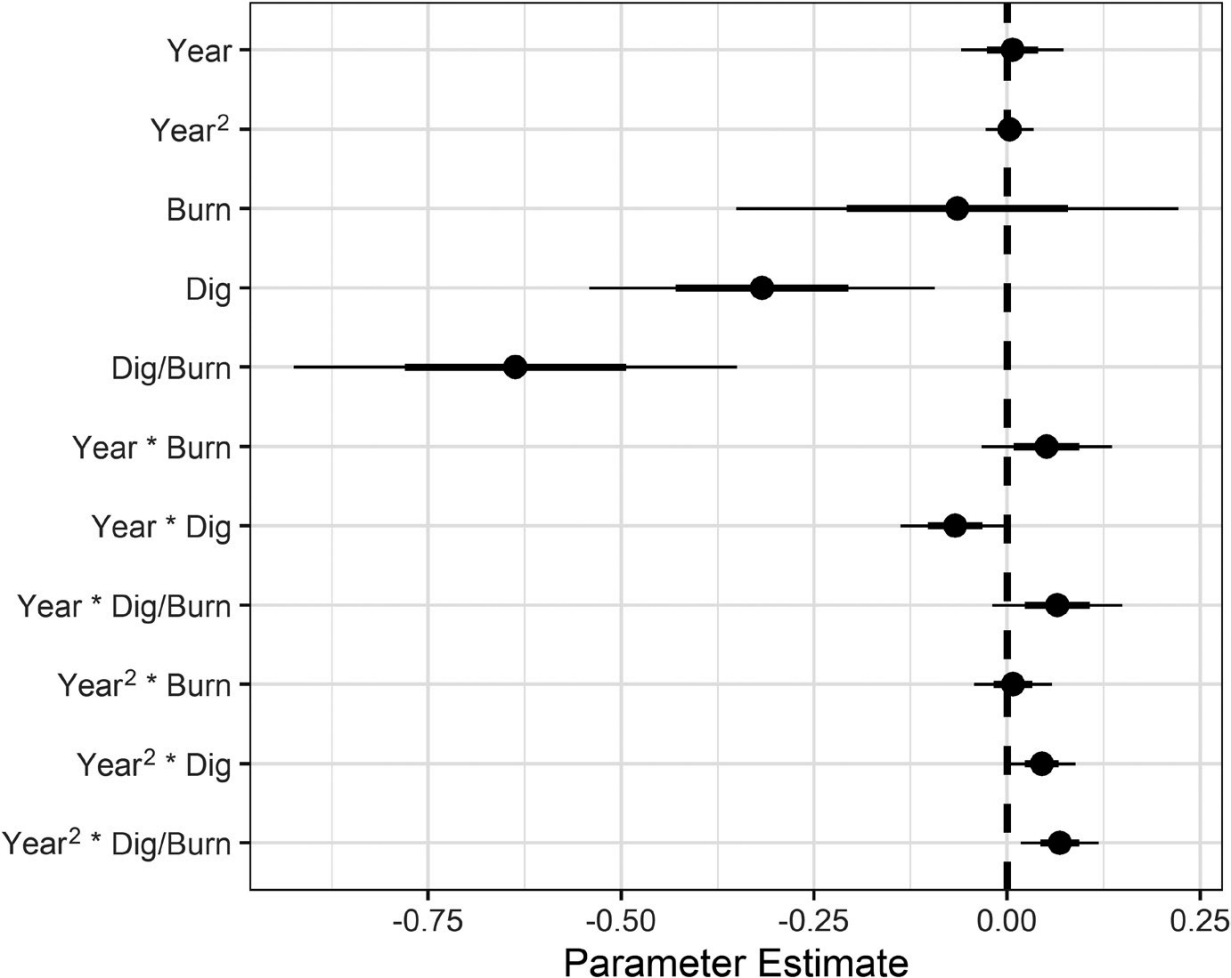
A coefficient plot showing 50th (bold line) and 95th (thin line) percentile uncertainty intervals obtained from generalized linear mixed effects model parameter estimates for single‐leaf camas plants. Effect sizes were strongest for dig and dig × burn treatment groups, relative to the control group. A modest positive trend over the study period in treated plots (Year^2^ × Dig, Dig/Burn) was observed, suggesting a tapering of the treatment effects over time (i.e., recovery)

We found strong evidence that digging only, and digging and burning in combination, substantially reduced single‐leaf camas abundance (Figure 4). Density of single‐leaved plants was 53% of control plots when harvested and burned (95% CI = 0.40, 0.70; *p* < .001) and 73% (95% CI = 0.58, 0.91; *p* = .005) when harvested. However, there was no meaningful difference between control and burned‐only plots (*p* = .653). We found an apparent tapering of the harvest treatment effect over time, revealed by the modeled interactions (105%, 95% CI = 1.00, 1.09, *p* = .039) and the harvest and fire treatment effect over time ($\bar{X}$ = 107%, 95% CI = 1.02, 1.13; *p* = .007), though we did not find any other treatment by time interactions (Table 1; Figure 4; Figure S1 and S2).

When back‐transformed (exp(*β*_0_) = 205 plants), the harvest treatment resulted in a net decrease, on average, of 55 single‐leaf camas plants (150, 95% CI = 119, 187; *p* = .005), the harvest and burning treatment resulted in an average net decrease of 96 single‐leaved plants (108, 95% CI = 82, 144; *p* < .001), and burning alone resulted in an average net decrease of only 12 single‐leaf plants (193, 95% CI = 146, 254; *p* = .653).

### 3.3 Treatment effects on multiple‐leaf camas plants

**FIGURE 5:**
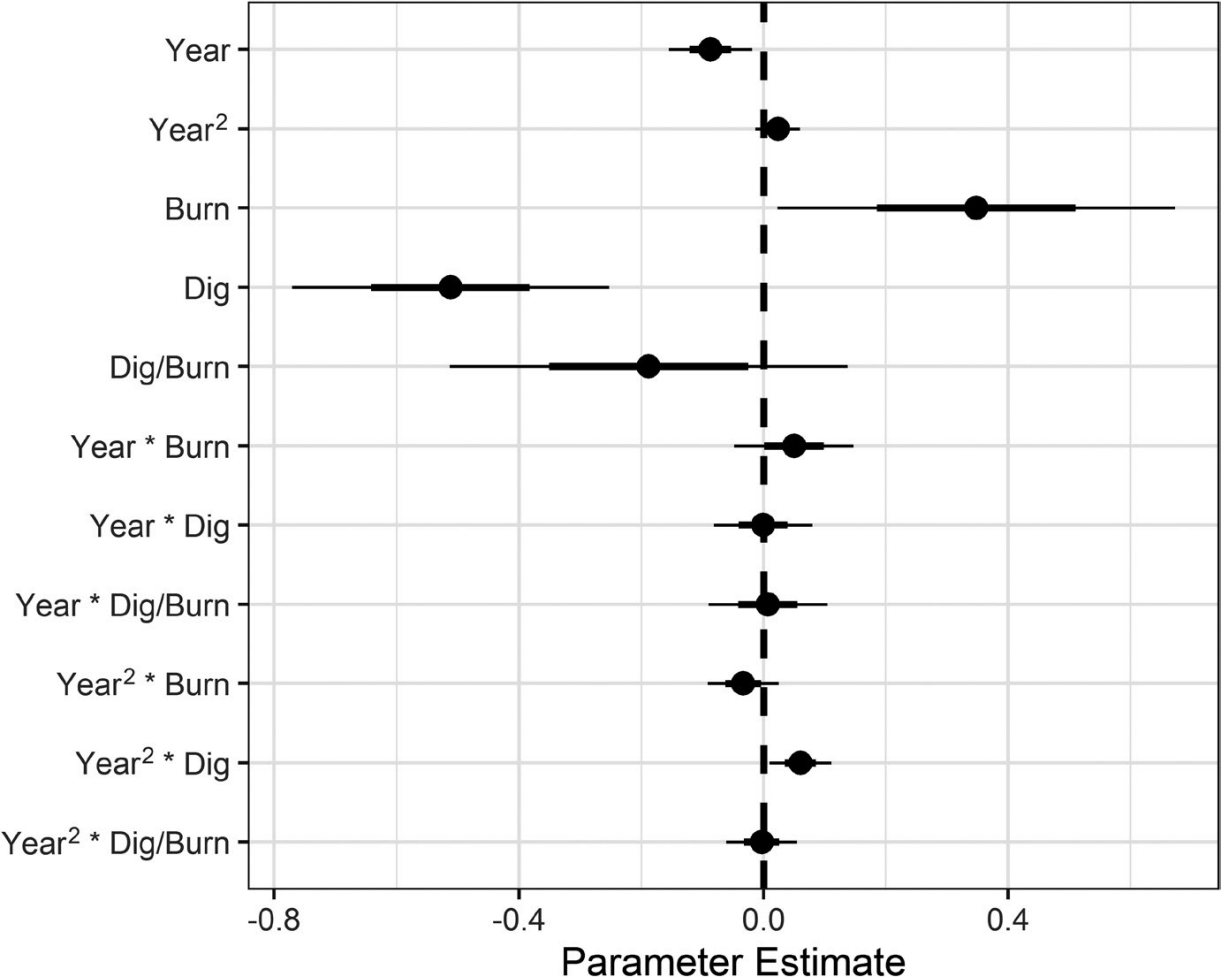
A coefficient plot showing 50th (bold line) and 95th (thin line) percentile uncertainty intervals obtained from generalized linear mixed effect model parameter estimates by treatment type for multiple‐leaf camas plants. Treatment effects were strong, relative to the control group, although uncertainty was higher than with effect size estimates on single‐leaf plants. A modest positive trend over the study period in dig‐only plots (Year^2^ × Dig) was observed, suggesting a tapering of that treatment over time (i.e., recovery)

We found an overall declining trend (92%) in the density of multiple‐leaf camas plants over the study period, irrespective of treatment (95% CI = 0.86, 0.98; *p* = .011; Table 1; Figure 5), but this did not obscure treatment effects. After accounting for this temporal trend, we found evidence that burning increased camas density, that digging reduced camas density, and that burning mediated the reduction from digging. Estimated density of multiple‐leaf camas plants was 142% of control plots when plots were burned (95% CI = 1.03, 1.95; *p* = .032) but only 60% of controls when harvested (95% CI = 0.46, 0.77; *p* < .001). Density of multiple‐leaf camas plants in plots that were both harvested and burned were lower (83%) than control but with an uncertainty interval that included 0 (95% CI 0.60–1.14; *p* = .249). As with counts of single‐leaf plants, we also found an apparent tapering of the harvest treatment effect on multiple‐leaf plants over time, revealed by the modeled interactions (106%, 95% CI = 1.01, 1.12, *p* = .017; Table 1; Figure 5; Figure S3 and S4).

When back‐transformed (overall average density of exp(*β*_0) =_ 68 plants), the harvest treatment resulted in a net decrease, on average, of 27 multiple‐leaf camas plants (95% CI = 31, 52; *p* < .001), whereas the harvest and burning treatment resulted in a net decrease in only 12 multiple‐leaf plants (95% CI = 41, 78; *p* = .249), and the burning treatment alone resulted in a net increase of 29 multiple‐leaf plants (95% CI = 70, 133; *p* = .032).

### 3.4 Treatment effects on flowering camas plants

**FIGURE 6:**
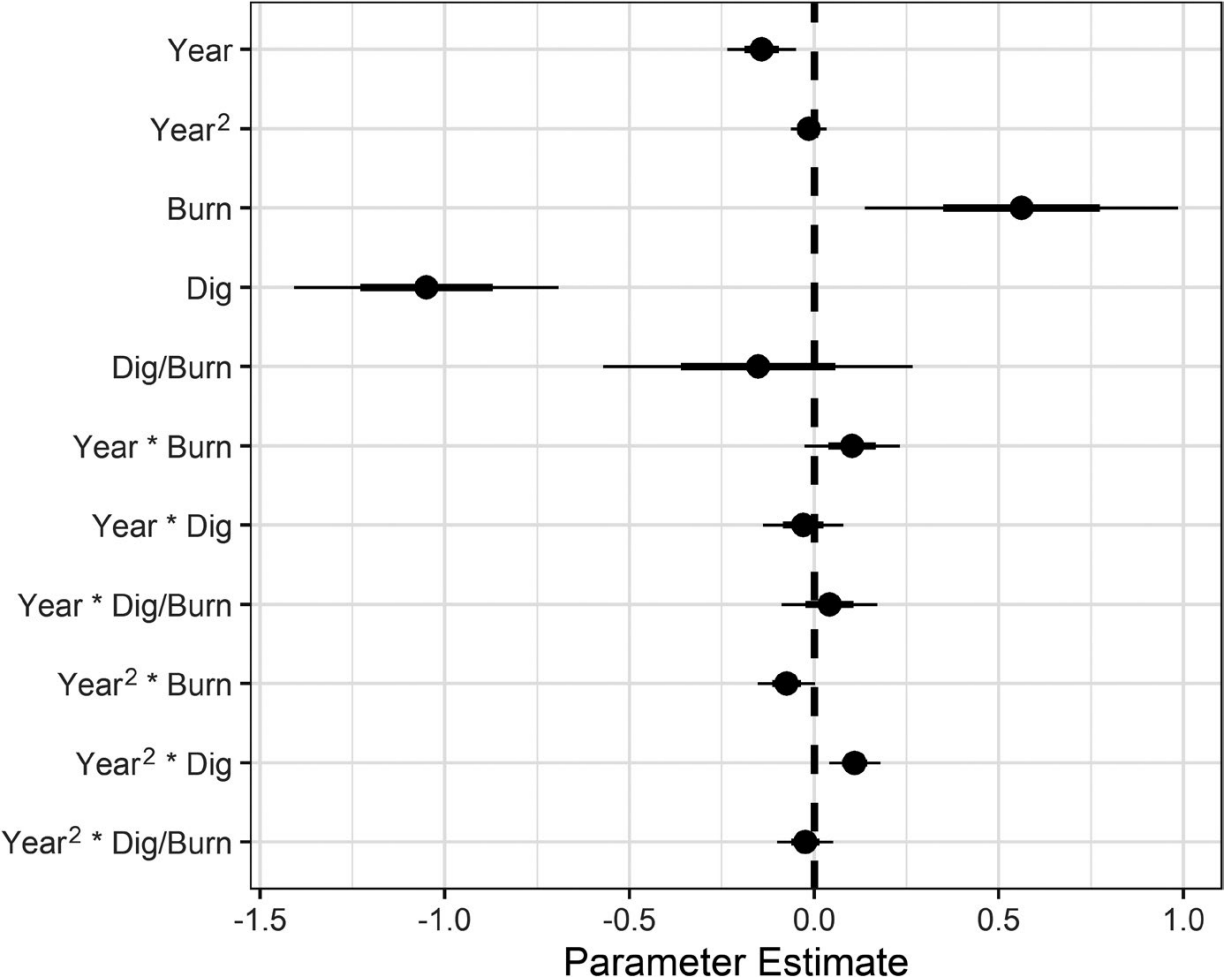
A coefficient plot showing 50th (bold line) and 95th (thin line) percentile uncertainty intervals obtained from generalized linear mixed effect model parameter estimates by treatment type for flowering camas plants. Treatment effects were strong, relative to the control group, and uncertainty to effect size estimates on multiple‐leaf plants. A modest positive trend over the study period in dig‐only plots (Year^2^ × Dig) was observed, suggesting a tapering of that treatment over time (i.e., recovery)

We found an overall declining trend (87%) in the density of flowering camas plants over the study period, irrespective of treatment (95% CI = 0.79, 0.95; *p* = .002; Table 1; Figure 6; Figure S5 and S6). After accounting for this trend, we found strong evidence that burning increased the abundance of flowering camas and mediated the reduction in flowering plant abundance caused by digging (Figure 6). Density of flowering camas plants was 175% of control plots when plots were burned (95% CI = 1.16, 2.66; *p* = .008), but just 35% of control plots when harvested (95% CI = 0.25, 0.50; *p* < .001). Plots that were dug and then burned maintained approximately that same net number of flowering plants (95% CI 0.57–1.30, *p* = .470).

As with single‐ and multiple‐leaf plants, we also found evidence that the negative effect of digging tapered over the course of the study (112%, 95% CI = 1.04, 1.19, *p* = .002), and also some evidence that the burn treatment effect tapered over time as well (93%, 95% CI = 0.86, 1.00; *p* = .056).

When back‐transformed (exp(*β*_0)_ = 14 plants), the harvesting treatment resulted in a net decrease, on average, of 9 flowering camas plants (5, 95% CI = 4, 7; *p* < .001), the burning and digging treatment resulted in a net decrease of 2 flowering plants (12, 95% CI = 8, 19; *p* = .470), but the burning treatment alone resulted in a net increase of 10.8 flowering plants (25, 95% CI = 17, 38; *p* = .008).

## 4 DISCUSSION

According to ethnobotanical literature from across the Pacific Northwest region of North America (Anderson, 1997; Suttles, 1951; Thoms, 1989), camas populations were thought to intensify in the presence of traditional harvest and land management activities. A series of observational studies have demonstrated beneficial effects of digging and burning (Anderson, 1997; Beckwith, 2004; Boyd, 1999; Hamman et al., 2011; Kramer, 2000; Stevens et al., 2000; Storm & Shebitz, 2006; Suttles, 1951; Thoms, 1989) and two unpublished experiments have also demonstrated such effects (Beckwith, 2004; Proctor, 2013). Our study strengthens the evidence for the hypothesis that these traditional resource management practices were beneficial to camas. Our study is unique by combining a before‐after‐control‐impact experiment with traditional ecological expertise that has direct application to protected area wetland management, including the potential resumption of traditional harvesting and burning activities, and provides an example for TEK/TRM studies, more broadly. We predicted that camas densities would increase within plots that were harvested using traditional methods and burned. Our results were consistent with these ideas to some extent but with notable exceptions. Traditional harvest reduced camas densities of all three life stage classes (single‐leaf, multiple‐leaf, flowering), whereas burning increased multiple‐leaf and flowering densities and ameliorated the reductions from digging. This latter finding that burning ameliorates removal of bulbs through digging is particularly noteworthy.

### 4.1 Effects of traditional harvest on camas

There was an initial reduction in multiple‐leaf and flowering camas plants in plots that were treated with traditional bulb harvest, reflecting the physical removal of plants. Single‐leaf camas plants also declined following the harvest treatment, possibly resulting from seeds being distributed at too great of depth, as camas seeds buried deeper than 1–2 cm will not successfully germinate (Watson, 1988). The harvest treatment effect tapered off for each of the three camas life stage classes as densities recovered during the study period. Declines in multiple‐leaf plant numbers following harvest were less than expected when compared with the numbers of bulbs removed during the first year of plot establishment and treatment, suggesting that smaller, unharvested camas bulbs are quickly able to recruit into larger plants by taking advantage of favorable growing conditions following harvest, including reduced competition and beneficial soil conditions.

### 4.2 Effects of fire on camas

Multiple‐leaf and flowering camas plant numbers were found to significantly increase following the burning treatment, results that are consistent with both anthropological research and contemporary experiments (Boyd, 1999; Hamman et al., 2011; Storm & Shebitz, 2006). Heating of organic matter in the soil may have resulted in an increase in the presence or availability of certain nutrients beneficial to camas growth (Anderson, 1993), though loss of nutrients due to volatilization is also possible (Blair, 1997; Knops et al., 2002), and competing effects may result in no overall change in soil nutrients (MacDougall & Turkington, 2007). The reduction in dense litter and thatch from pasture grasses was apparent following burning and likely provided camas plants greater access to light during spring emergence. Single‐leaf camas plants appeared to decrease following the burning treatment, possibly a result of heat damage to the seeds situated near the surface, though declines in single‐leaf plants were not statistically significant.

### 4.3 Effects of combined harvest and fire on camas

Multiple‐leaf and flowering camas plant declines caused by bulb removal during harvest were ameliorated by applied fire following harvest. Notably, the addition of fire to harvested plots further reduced counts of single‐leaf camas plants but actually yielded a net increase in multiple‐leaf and flowering densities relative to harvested densities. This discrepancy in outcome among the life stage classes may have occurred if some seed were buried too deeply in the soil following refilling of the harvested plot while other seeds that remained near the surface were destroyed by the heat of the fire. In contrast, the bulbs of larger plants not removed during harvest may be able to survive the short‐duration heating from fire, as it was applied in our study. The action of removing competitive vegetation and releasing nutrients previously noted may also stimulate a sufficient compensatory increase in bulb germination and flowering to explain the observed amelioration.

### 4.4 Effects of time since treatment on camas

The disturbances associated with traditional bulb harvest are pronounced and appear to be long lasting in some instances, though the ultimate effects of harvest and fire on camas populations in our experiment may not be realized for many years due to the slow development and long lifespan of individual plants. Interestingly, and despite the sizeable declines and strong treatment effects first observed following the harvest and combined harvest and burning treatments, we found consistent tapering of treatment effects on densities of each of the three life stage classes over the 5–6 years of post‐treatment study indicating that camas plant densities within plots were rebounding to pre‐treatment levels. The burning treatment effect also tapered over the study period indicating that the positive effects introduced by burning are also temporary. Overall declining camas plant counts, as evidenced by the control plots, were consistent with the results of site‐wide camas monitoring efforts (NPS, unpublished data) and may reflect plant dormancy caused by unfavorable growing conditions over the course of the study period that were not captured in our models. Further study of the relationship between wild common camas plants and climate, including the long‐term interaction of camas plants with temperature and precipitation, is necessary to understand these longer‐term dynamics of camas populations.

### 4.5 Uncertainty arising from dormancy and overdispersion

There are difficulties in studying a geophyte that exhibits routine dormancy that may actually vary over time in response to harsh growing conditions and density‐dependent responses to harvest. Dormancy is a likely explanation for our surprising finding that observed counts of plants in some harvested plots exceeded the preharvest (i.e., before impact) counts. Dormancy has been shown to be a common trait in many species of geophytes, including some species of camas (Beckwith, 2004). Our study reveals the necessity of quantifying dormancy length and frequency in camas plants, as well as the influences on dormancy from selective removal during harvesting and changes to climate or the duration of standing water during spring growth. Geophyte dormancy will be particularly important for understanding long‐term changes and forecasting population persistence under scenarios of accelerated climate change, for example. This will be requisite for the establishment of sustainable harvest plans. Due to the length of time required for camas to reach maturation (3–5 years from seed germination to flower development; Bailey [1914]; Genders [1973]; Thoms [1989]) and the reported longevity of the plant (20–30 years; Thoms [1989]), a significant period of study will be required.

Overdispersion in camas populations presents another challenge for quantifying the impacts of traditional resource management practices or addressing other questions such as long‐term climate change impacts. Overdispersion in our study arose from several sources, including dormancy, but also microtopography and drainage patterns (Rodhouse et al., 2011), and influenced our decision to employ the negative binomial mixed‐effects model. Although overdispersion is common in many ecological studies, we suspect it will be particularly characteristic of TEK/TRM studies involving indigenous plant foods that must have occurred in at least local (hence patchy) abundance in order to have supported human populations.

### 4.6 Management implications

Common camas is an ecologically important plant that has been historically widespread in wet prairies across the Pacific Northwest but has become considerably rarer as wetlands have been drained and converted to agricultural landscapes. New threats are emerging from accelerated climate change and drought. Careful management will be necessary to maintain camas productivity in order to support both wildlife and traditional harvest. Camas leaves and flowers are reportedly eaten by ungulates (Craighead et al., 1963), and small mammals including voles and pocket gophers will eat and even store the bulbs (Stevens et al., 2000; D. Stucki, pers. obs.). Camas plants are host to a variety of native insect pollinators that are necessary for camas seed production (Bartow, 2015; Pendergrass et al., 2008), and although camas plants are capable of reproducing vegetatively, sexual reproduction is far more common (Thoms, 1989). Reductions in seed production may limit regeneration and result in further reductions of camas numbers. Plant declines would likely have a negative impact on pollinator populations, potentially resulting in further declines of other insect pollinated plants within these wet prairie ecosystems.

The continuation or reintroduction of traditional harvest and resource management may emerge as a viable strategy in many remnant camas prairies. A sustainable harvesting return interval of approximately 5 years has been proposed in the anthropological literature (Thoms, 1989) and previous field studies (Beckwith, 2004). During our study period, camas plant densities rebounded substantially following harvest, and baseline counts were surpassed in some instances, though declines were also seen (see Figures S1‐S6). Because harvest produced a negative treatment effect in some plots 5 years later in our study, we recommend that harvest of a particular site should occur no more frequently than 5 years in a similar climate and system and that a sustainable interval may be longer than 5 years depending on prevailing climatic conditions during previous years, that is, extended drought or above‐average temperatures. Fire appeared to improve the negative effects of harvest and may be an important component of management for a sustainable harvest. As with any situation where a natural resource is being extracted, it is important to avoid harvesting more frequently than the landscape can support. Noxious weed invasion is also a risk when causing soil disturbance, and harvesting sites should be monitored for emerging plant infestations.

The experimental methods used in our study, informed by TEK specialists, in combination with a broader site‐wide camas population monitoring program (e.g., Rodhouse et al., 2007), can provide the real‐time feedback necessary to evaluate both the return of traditional practices and other influences such as climate change and to conduct adaptive management. It furthermore represents a novel way to bridge the gap between contemporary quantitative ecological science and TEK and facilitate adherents of both paradigms to meaningfully collaborate and learn from one another in pursuit of common conservation goals. Camas has evolved along with significant human interaction over a span of thousands of years and understanding the anthropological impacts on camas populations is necessary to enable holistic wet camas prairie restoration and conservation. Traditional resource management techniques remain a mostly untapped toolset for land managers when developing restoration strategies that are culturally and ecologically relevant to an area. Evaluating TRM methods will be instrumental toward identifying appropriate restoration strategies that balance traditional ecological knowledge with efficient and effective land management in a modern landscape.

## CONFLICT OF INTEREST

None declared.

## AUTHOR CONTRIBUTIONS

**Devin S. Stucki:** Conceptualization (equal); Formal analysis (equal); Investigation (lead); Methodology (lead); Writing‐original draft (lead); Writing‐review & editing (equal). **Thomas J. Rodhouse:** Conceptualization (equal); Formal analysis (equal); Investigation (supporting); Methodology (supporting); Writing‐original draft (supporting); Writing‐review & editing (equal). **Ron J. Reuter:** Methodology (supporting); Writing‐original draft (supporting); Writing‐review & editing (equal).

## Supporting information

Figure S1

Figure S2

Figure S3

Figure S4

Figure S5

Figure S6

Supplementary Material

Supplementary Material

## Data Availability

Data associated with this experiment can be found at the National Park Service IRMA repository (https://irma.nps.gov/DataStore/Reference/Profile/2286897).
