# Supplementary figures and images for: Effects of traditional harvest and burning on common camas (Camassia quamash) abundance in Northern Idaho: The potential for traditional resource management in a protected area wetland
Source: Ecol Evol. 2021 Sep 1;11(23):16473–86. doi: 10.1002/ece3.8010 (PMC8668748; doi:10.1002/ece3.8010)

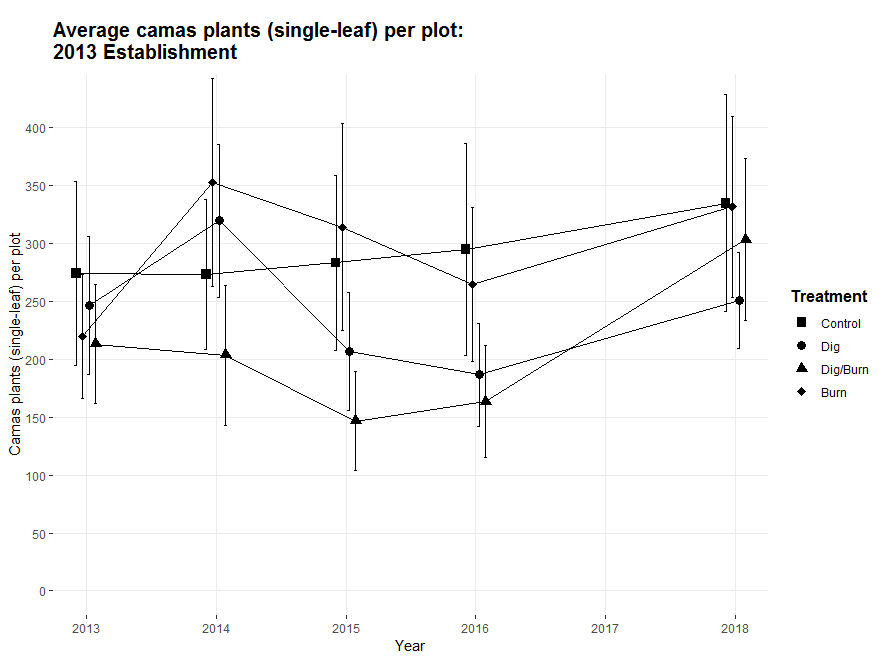

Supplement: Supplementary file 1 — Figure S1 [file ECE3-11-16473-s006.tiff]

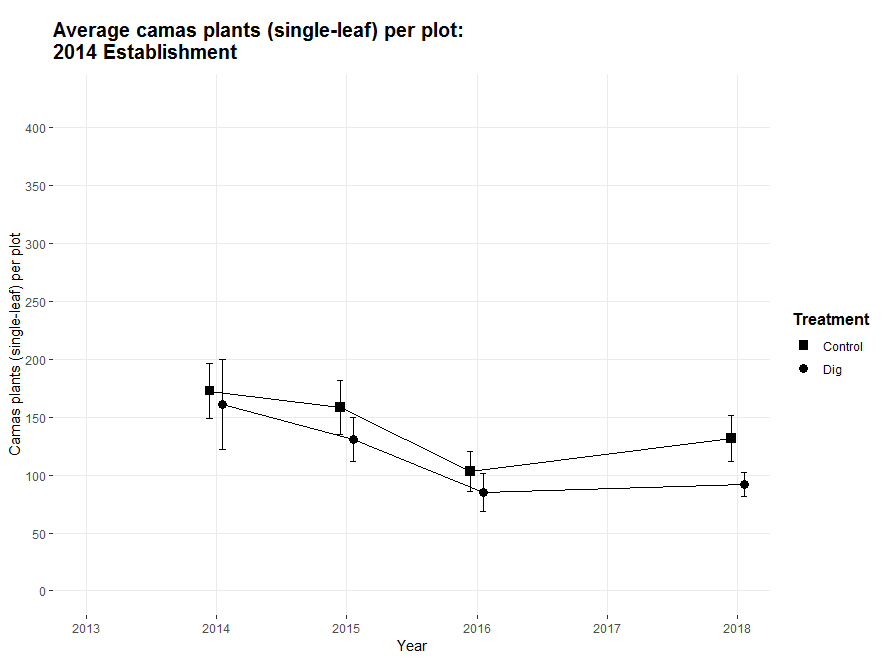

Supplement: Supplementary file 2 — Figure S2 [file ECE3-11-16473-s005.tiff]

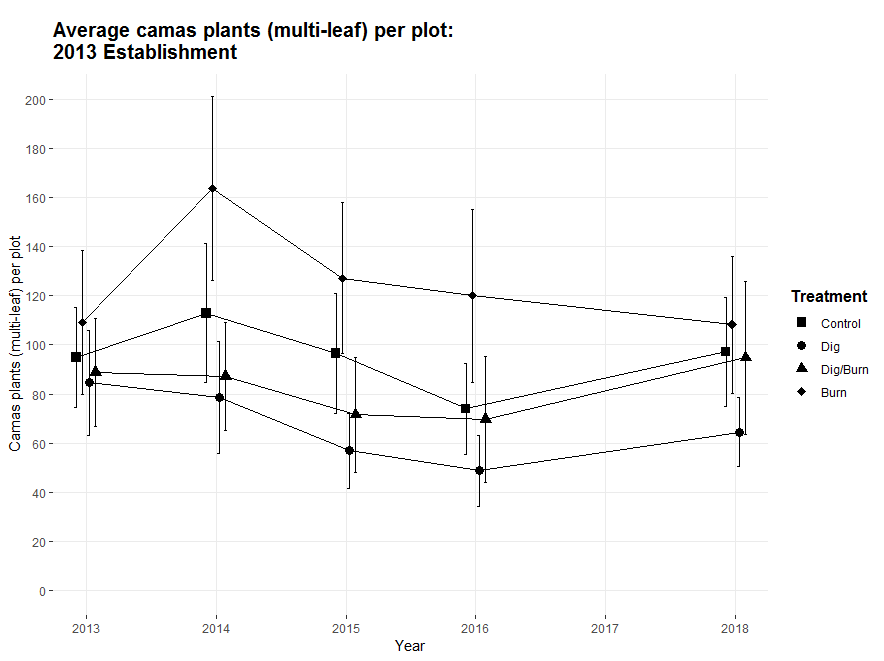

Supplement: Supplementary file 3 — Figure S3 [file ECE3-11-16473-s007.tiff]

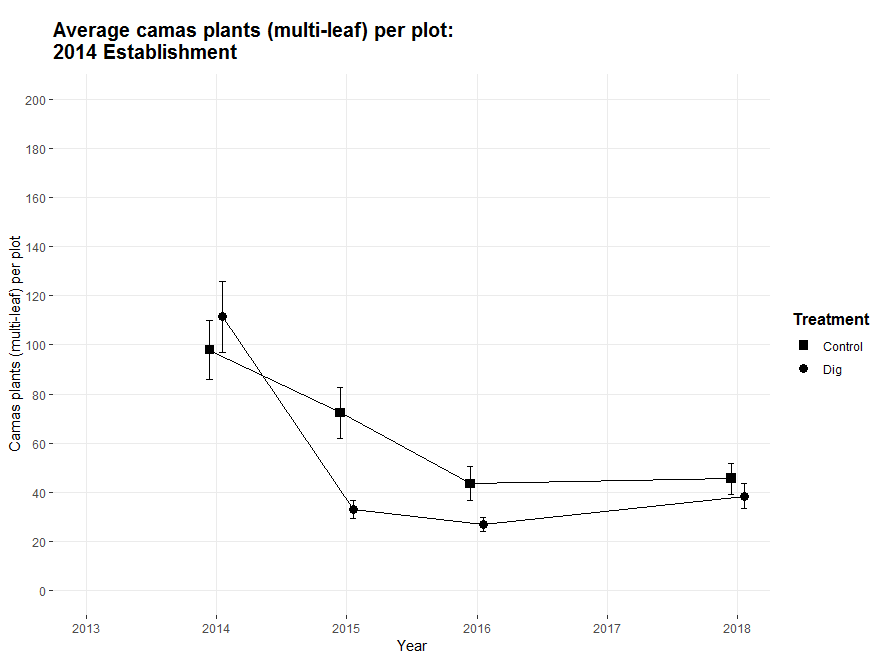

Supplement: Supplementary file 4 — Figure S4 [file ECE3-11-16473-s003.tiff]

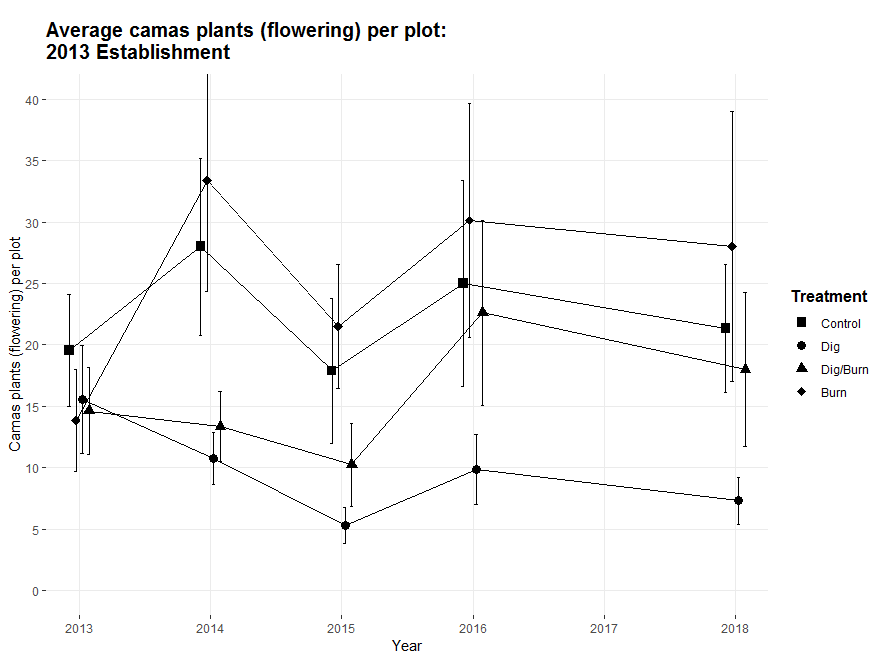

Supplement: Supplementary file 5 — Figure S5 [file ECE3-11-16473-s001.tiff]

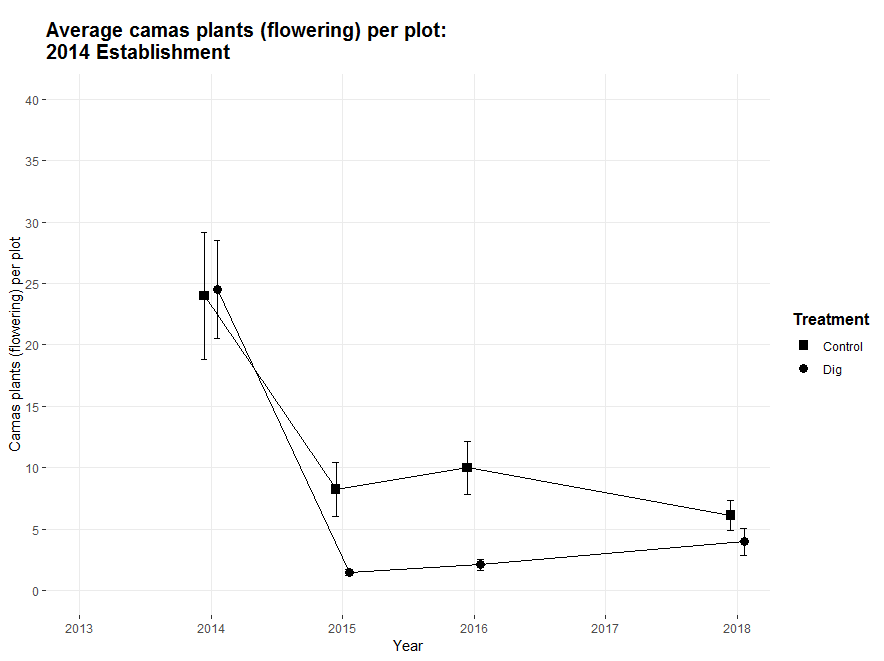

Supplement: Supplementary file 6 — Figure S6 [file ECE3-11-16473-s004.tiff]
