# Supplementary material for: Effects of traditional harvest and burning on common camas (Camassia quamash) abundance in Northern Idaho: The potential for traditional resource management in a protected area wetland
Source: Ecol Evol. 2021 Sep 1;11(23):16473–86. doi: 10.1002/ece3.8010 (PMC8668748; doi:10.1002/ece3.8010)
Supplement: Supplementary file 7 — Supplementary Material [file ECE3-11-16473-s002.docx]

Supplementary materials S1

Camas Bulb Digging Pilot Data Collection Notes

*Camas digging with* *Lee Bourgeau, Kamelle Bourgeau, Diane Mallickan, Vivian Wilson, and Jannis Jocius on Monday, 10 September 2012.*

We drove to the Weippe site and I decided to go to section A—the area of densest aboveground camas vegetative density (using 2011 and prior camas monitoring data). Just past the ditch between sections A and B, we dropped our things and took out our digging implements. The park staff had tukuses made with wooden handles and curved metal stakes about 2.5 feet long. Lee and Kamelle had brought 2 spading forks (potato forks) to use and said they were more efficient at moving soil. As a side note, I heard that other Tribal members only use tukuses. Lee did bring a tukus, but never used it.

Diane and I asked to watch Lee and Kamelle since we had never actually dug with any seasoned root-diggers before. Essentially, Lee would find a spot with lots of dried camas stalks and use her fork (pushing at an angle with her foot on the top of the tines) to loosen ground in a circular fashion—to create an (approximately) 3 foot diameter hole. She dug about 6-8 inches deep.

Then, Lee sat on the ground and used her gloved hands to break apart densely compacted soil to extract the large camas bulbs. The bulbs she kept were about the size of the width of her pointer finger or bigger (about 1.5 cm across, or larger). Lee and Kamelle both had hand-sewn baskets of cloth and twine/rope connected to a long strap of cloth strapped around their waists for easy camas collection (just dig, rub off the soil, and drop in the bag!). The rest of the staff used burlap sacks bought at Big R, and these worked fine. They allowed for the bulbs to “breathe” and dry out (a plastic bag would promote mold and perspiration).

As Lee worked, she would enlarge her original hole as she saw more bulbs peeking out of the wall of the hole so that her 3-inch diameter hole would change shape—get longer or wider. Once the camas was harder to find, she would put all of the soil back into the hole and move on to another nearby (about 3 feet away or so) spot to dig again.

All of the camas bulbs 1.5 cm or smaller were either not worth the bother to be taken out of the dirt clods, or were thrown to the side to be put back in the hole once Lee was finished with it.

When Lee put the soil back in the pit, she sort of replaced the O horizon where it used to be—that is, the duff, nonvascular plants, grasses, and roots near the surface of the soil were placed on the top of the filled hole. This was not always the case though. In other words, you could tell a hole was dug because the sod was not replaced exactly as it was extracted—mostly because some of the bulbs were right below the soil surface and some were 15-20cm (6-8 inches) deep—which meant the top sod was not kept fully intact.

Lee did not sprinkle seeds into the hole, but the seeds probably fell in anyway because, this late in the season, the seed pods are open and digging and knocking plants over naturally made the seeds fall to the ground and get soil to seed contact.

Sometimes Lee stepped on the area just dug and covered to even out the soil.

I noticed the soil was very dry (dusty) near the soil surface. This was the same layer of soil that was packed full of perennial and annual grass and forb roots. The root layer only extended about 8 cm (3 inches) down. Below that was a denser layer of soil—somewhat moist to the touch, darker in color, and riddled with insect galleries (worm-type borrowings). I did not see hardly any insects in the soil profile.

I asked Lee whether the bulbs here were any smaller than those found at Mussellshell meadows (where she and Kamelle normally dig). She said no. I asked whether the moisture in the soil at this time of year was any different. She said yes. It is pretty obvious when you visit each site at this time of year anyway. Overall, we spent about 3 hours digging at the site. All the park service staff pooled their camas bulbs together. We did not count or measure them on this trip. Lee said that the amount of camas she dug in the amount of time we had was equivalent to what she usually digs in her traditional digging spots.
