# Supplementary material for: Effects of traditional harvest and burning on common camas (Camassia quamash) abundance in Northern Idaho: The potential for traditional resource management in a protected area wetland
Source: Ecol Evol. 2021 Sep 1;11(23):16473–86. doi: 10.1002/ece3.8010 (PMC8668748; doi:10.1002/ece3.8010)
Supplement: Supplementary file 8 — Supplementary Material [file ECE3-11-16473-s008.docx]

Supplementary materials S2


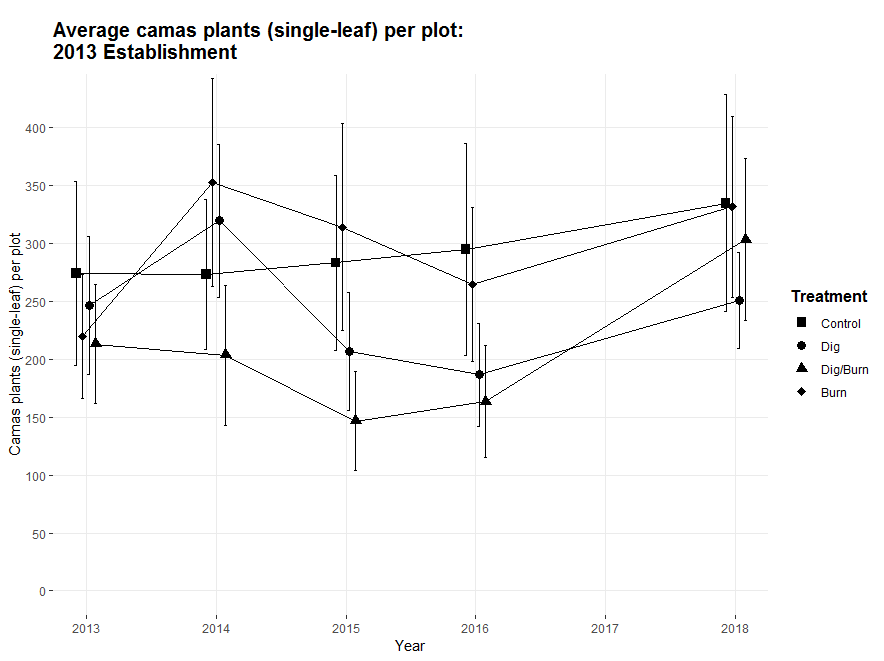


Figure S1. Average number of single-leaf camas plants per plot established in 2013, organized by treatment type. Plot points and standard error bars are jittered to allow for easier viewing of overlapping symbols.


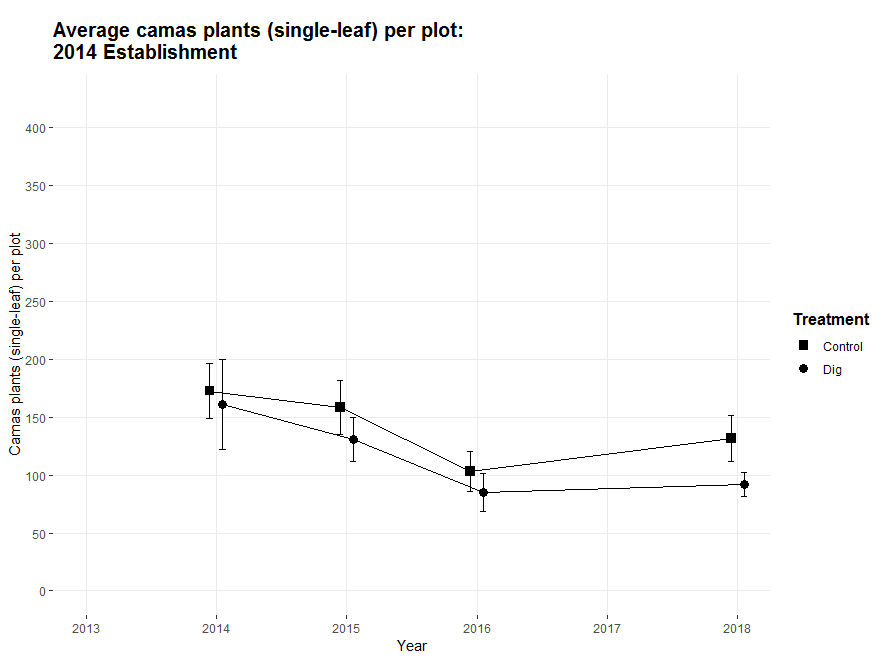


Figure S2. Average number of single-leaf camas plants per plot established in 2014, organized by treatment type. Plot points and standard error bars are jittered to allow for easier viewing of overlapping symbols.


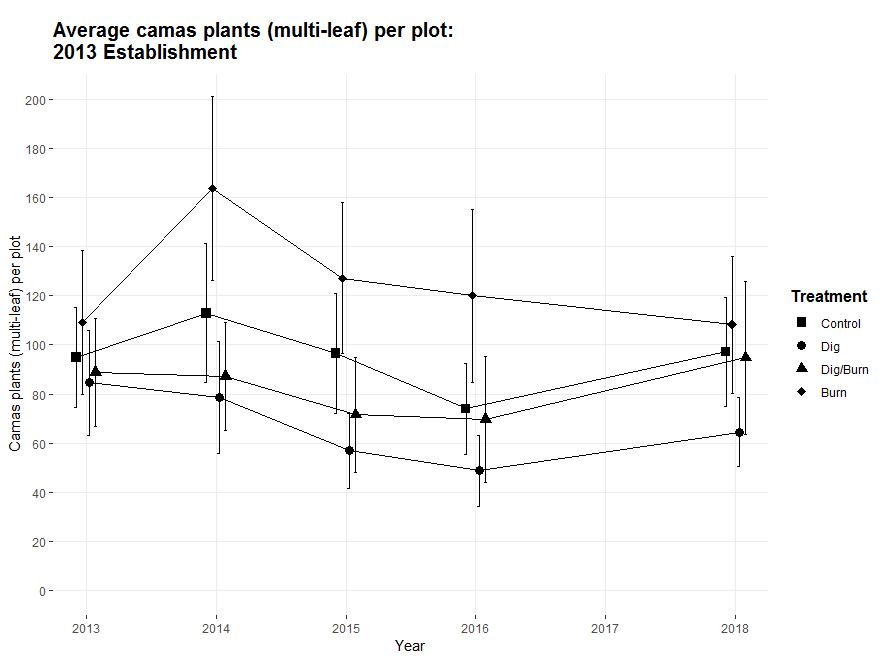


Figure S3. Average number of multiple-leaf camas plants per plot established in 2013, organized by treatment type. Plot points and standard error bars are jittered to allow for easier viewing of overlapping symbols.


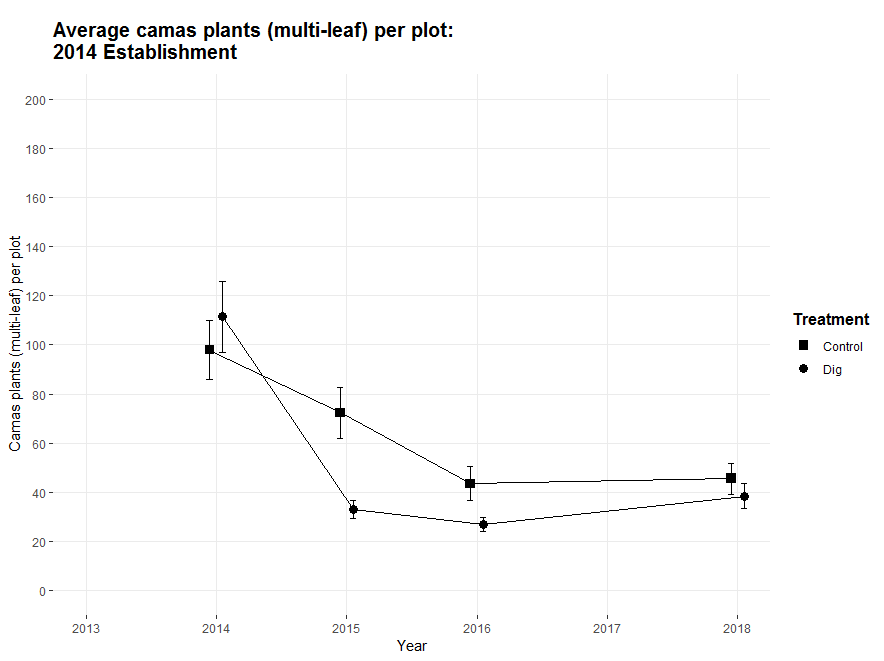


Figure S4. Average number of multiple-leaf camas plants per plot established in 2014, organized by treatment type. Plot points and standard error bars are jittered to allow for easier viewing of overlapping symbols.


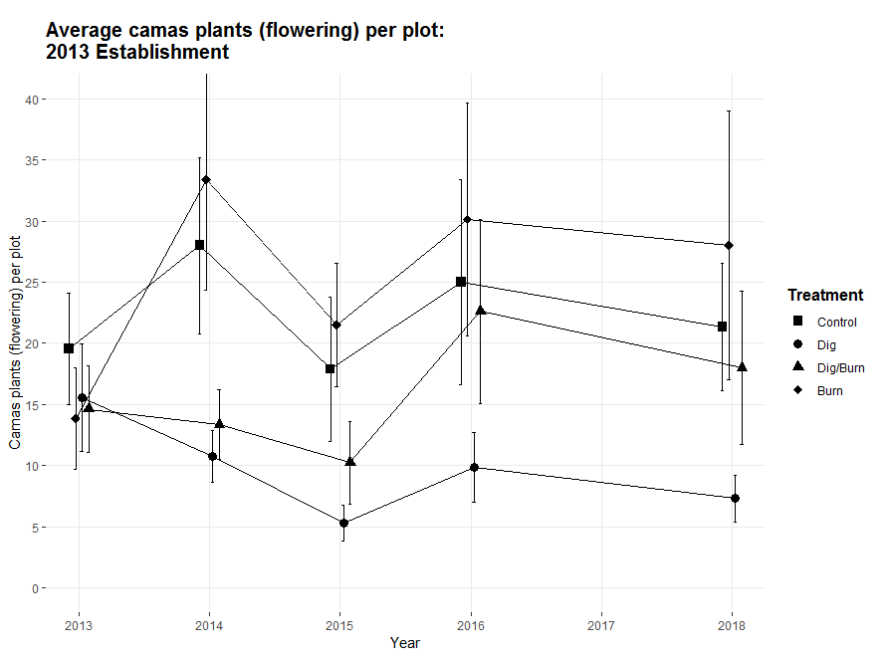


Figure S5. Average number of flowering camas plants per plot established in 2013, organized by treatment type. Plot points and standard error bars are jittered to allow for easier viewing of overlapping symbols.


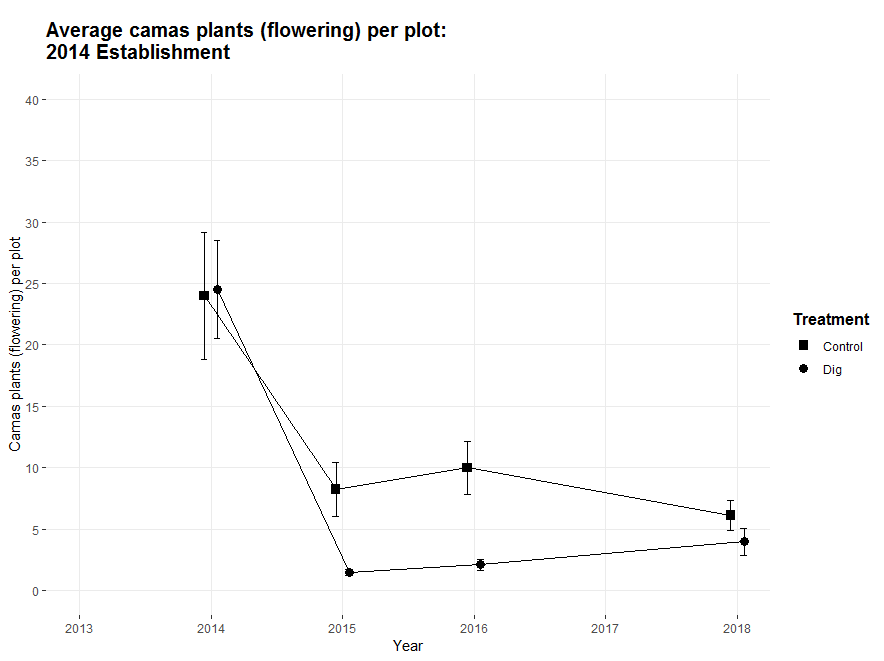


Figure S6. Average number of flowering camas plants per plot established in 2014, organized by treatment type. Plot points and standard error bars are jittered to allow for easier viewing of overlapping symbols.
